# Evaluating educational performance and achievements of faculty in general medicine departments of Japanese universities

**DOI:** 10.1002/jgf2.537

**Published:** 2022-03-17

**Authors:** Masaki Tago, Kiyoshi Shikino, Takashi Watari, Risa Hirata, Shun Yamashita, Yoshinori Tokushima, Midori Tokushima, Naoko E. Katsuki, Motoshi Fujiwara, Shu‐ichi Yamashita

**Affiliations:** ^1^ 469771 Department of General Medicine Saga University Hospital Saga Japan; ^2^ Department of General Medicine Chiba University Hospital Chiba Japan; ^3^ General Medicine Center Shimane University Hospital Shimane Japan

## Abstract

A cross‐sectional questionnaire‐based study was conducted to examine whether the educational performance and achievements are appropriately recognized and contribute to their promotion in university hospitals. We found that the chairpersons of those general medicine departments believed that educational performance had not been appropriately evaluated; educational achievements did not receive sufficient consideration for promotion compared with the performance and achievements related to clinical and research activities.

To the Editor,

Medical education is essential for developing the academic field of general medicine.^1^ In general medicine, teachers are naturally responsible for such education. To promote their willingness to make a contribution to education, the educational performance and achievements of university faculty members need to be fairly and fully evaluated with respect to promotion.^2^ However, their educational performance and achievements may not be adequately recognized and may not contribute to their promotion in university hospitals.^3^

We conducted, therefore, a cross‐sectional questionnaire‐based study to examine that issue. On June 28, 2021, we sent questionnaires to all 82 universities on the public mailing list of the Council of Japanese University Hospitals for General Medicine. That council covers general medicine departments in Japan’s university hospitals; its annual meeting aims to promote communication and information sharing among such institutions. Respondents answered each question using a 6‐point Likert scale (from 1 [strongly disagree] to 6 [strongly agree]) to investigate whether the performance in clinical practice, research, and education was adequately evaluated in those university hospitals. We also surveyed the importance of clinical, research, and educational achievements for promotion at the universities, with a total score of 100 points (e.g., clinical achievement 40, research achievement 20, and educational achievement 40). The data were collected using an online platform. The department chairperson was responsible for completing the questionnaire, which required the department name and chairperson’s position to be stated.

Among 71 universities with a general medicine department, 46 responded (response rate, 64.7%). We analyzed the responses from 43 universities with no missing data. We found that the chairpersons of those general medicine departments believed that educational performance had not been appropriately evaluated; educational achievements did not receive sufficient consideration for promotion compared with the performance and achievements related to clinical and research activities (Table 1). Regarding performance, we defined a Likert score of 4 or more as a positive response and that of 3 or less as a negative response. The positive and negative responses were 23 and 20 for clinical performance, 27 and 16 for research performance, and 18 and 25 for educational performance, respectively. Thus, positive responses for educational performance were the lowest among all three categories.

**TABLE 1:** Survey results about evaluating educational performance and achievements at Japanese universities

Performance adequately evaluated^a^	Mean (±standard deviation)
Clinical performance	3.6 ± 1.1
Research performance	4.0 ± 1.0
Educational performance	3.2 ± 1.2
**Importance of achievements for promotion** **b**	**Median (interquartile)**
Clinical achievement	30 (20–34)
Research achievement	40 (40–60)
Educational achievement	20 (10–30)

Questions answered on a 6‐point Likert scale (from 1 [strongly disagree] to 6 [strongly agree]).

Total score for the importance of achievements was 100.

This study showed that the educational achievement and performance of faculty members in general medicine departments at Japan’s universities were not adequately assessed. Fostering a greater number of academic generalists who can provide proper education to the next generation of general medicine practitioners will help develop the field.^4^ Quality education would encourage more medical students to study general medicine.^5^ Thus, the educational achievements of faculty members in departments of general medicine at Japan’s universities merit adequate assessment, which could help develop that medical field. Teaching portfolios are helpful for evaluating educational activities^6^; however, no well‐established model for evaluating educational activities has yet been developed, and this does not apply only to the field of general medicine. Thus, further research is required in this regard.

## CONFLICT OF INTEREST

The authors have stated explicitly that there are no conflicts of interest in connection with this article.
